# A cross-sectional study design to determine the prevalence of knowledge, attitude, and the preventive practice of food poisoning and its factors among postgraduate students in a public university in Selangor, Malaysia

**DOI:** 10.1371/journal.pone.0262313

**Published:** 2022-01-28

**Authors:** Arhyel Buba Mshelia, Malina Osman, Norashiqin Binti Misni

**Affiliations:** 1 Department of Veterinary Public Health and Preventive Medicine, Faculty of Veterinary Medicine, University of Maiduguri, Borno State, Nigeria; 2 Department of Medical Microbiology, Faculty of Medicine and Health Sciences, Universiti Putra Malaysia, Serdang, Selangor, Malaysia; Guru Angad Dev Veterinary and Animal Sciences University, INDIA

## Abstract

**Objectives:**

This study is designed to determine the knowledge, attitude, and practice of food poisoning and its factors among postgraduate students in Universiti Putra Malaysia.

**Methods:**

A cross-sectional study was conducted among 212 respondents who were selected through simple random sampling. Data was generated using a validated and reliable self-administered questionnaire.

**Results:**

The majority of the respondents are male, aged less than 35 years old, non-Malaysian, single, first-degree holders, not working, received a monthly income of less than 3264 Malaysian Ringgit, aware of food poisoning outbreak and the sources of their information of the food poisoning outbreak were television, the internet, newspaper, Online journals, friends, Facebook, community, nurse, drinking raw milk for the second time, information from their parents, relatives, restaurant, and radio. Majority had previous history of food poisoning illness but didn’t correctly confirm the causes of their food poisoning illness. Majority had poor knowledge, acceptable attitude, and good practice of food poisoning. A significant association was observed for citizen, marital status, awareness of food poisoning outbreak, and previous history of food poisoning illness with knowledge. Gender and awareness of food poisoning outbreak were significantly associated with attitude. Attitude and practice were significantly associated. Logistic regression revealed that being married, awareness of food poisoning outbreak, and previous history of food poisoning illness are predictors for good knowledge. Female respondents and awareness of food poisoning outbreak are predictors for acceptable attitude.

**Conclusion:**

Documentation of the identified poor knowledge and factors affecting knowledge, attitude, and practice provides essential information on the baseline indicators towards the risk of food poisoning among the respondents. A relevant interventional program is highly recommended to prevent the potential risks of food poisoning outbreak among them.

## Introduction

Food poisoning is a disease caused by the consumption of food or water contaminated with bacteria and/or their toxins, chemicals, viruses, or parasites. Improper food or drink handling, production, or storage usually bring about food contamination [1]. The prevention of food poisoning requires applying food hygiene and food safety as vital components and also educating consumers on the choices of food premises [2, 3]. knowledge is the capacity to gain, remember, and use information; a combination of understanding, experience, ability to judge well, and skill. Attitude refers to the tendency or readiness to respond in a positive way to specific circumstances, to see and interpret events according to certain predispositions, or to organize opinions or beliefs into well-organized interrelated structures. Practice means the application of rules and knowledge that leads to action. A good practice is an art that is related to the advancement of knowledge and applied science and is achieved in an ethical manner [4].

Various studies have found either poor knowledge, attitude, or practice towards food poisoning; or in some cases, both poor attitude and practice among respondents [5–13]. Roughly 600 million, almost 1 in 10 population worldwide, fail ill after consuming contaminated food, and 420 000 die every year, giving rise to the loss of 33 million healthy life years (DALYs). Unsafe food presents a global health risk putting everyone at risk. Infants, young children, pregnant women, the elderly, and those with an underlying illness are highly susceptible. Every year 220 million children develop food poisoning, and 96 000 dies. Unsafe food produces a vicious cycle of diarrhea and malnutrition, risking the nutritional status of the most susceptible [14].

The risk of food poisoning is most serious in low- and middle-income countries. And it is related to unsafe water, poor hygiene, poor conditions of producing and storing food, lower level of literacy and education, and inadequate food safety legislation or the establishment of such legislation. Food poisoning disease can cause short-term symptoms like nausea, vomiting, and diarrhea. Nevertheless, it can also give rise to longer-term illnesses like cancer, kidney failure or liver damage, brain and neural disorders. After the African region, the World Health Organization South-East Asia Region has the second-highest burden of food poisoning diseases per population. But concerning absolute numbers, more populations living in Southeast Asia Region fall ill and die from food poisoning diseases every year than in any other World Health Organization Region, with more than 150 million episodes and 175 000 deaths annually [15].

For the Asian countries apart from Japan in comparison, not much has been performed on the surveillance of food poisoning. Almost all data are obtained from particular yet inadequate investigations and studies. The incidence of food poisoning in Malaysia was 9.62 per 100,000 population in 1981, with the regularly isolated etiological agents being *Staphylococcus aureus*, *Vibrio parahaemolyticus*, and *Salmonella species* [16]. In addition, there is an underestimation of food poisoning because the affected populations do not communicate nearly all cases, and many processes are required before the case is brought to the authority. Only a few cases of food poisoned individuals seek medical attention at clinics or hospitals. As a result, only these few persons are reported to the public health authorities [17].

The incidence rate of food poisoning significantly escalated from 2005 to 2013, which accounted for some mortality [17]. In 2016, Selangor turned out as the state with the highest reported cases of food poisoning, followed by Kedah, Perak, and Kelantan [18]. The incidence rate of food poisoning from 2012–2019 shows a decreasing and increasing secular trend [19–26]. Food service outlet in universities is the major dining platform for students and give rise to a high dependency on food sold on campus. But cases of food poisoning in Malaysia are still happening in universities and colleges because of inappropriate practices among food handlers. The worry emerges when students are exposed to the risks of food poisoning illness [27].

There is a lack of previous studies to evaluate the knowledge, attitude, and preventive practice of food poisoning among students in Universiti Putra Malaysia. University students are a group of population that confront many risks because of their unsafe food consumption practice. There were unsatisfactory findings on young adult students concerning knowledge, attitude, and practice because of an incompetent study designed on acceptably large population size for the age group. As a result, finding ways to deliver better education and lower food poisoning illness is to have an endless understanding of young students’ knowledge, attitude, and practice about food safety [10].

This study aims to determine the characteristics of the respondents based on demographic (Age, gender, and citizen) and socioeconomic factors (educational level, monthly income, marital status, employment status); level of knowledge; attitude; and the preventive practice of food poisoning; awareness of food poisoning outbreak; source of information of the outbreak; previous history of food poisoning illness; the cause of the food poisoning illness; association between the respondents’ sociodemographic factors, awareness of food poisoning outbreak, and previous history of food poisoning illness with knowledge, attitude, and preventive practice of food poisoning; association of knowledge and attitude with the preventive practice of food poisoning; and predictors influencing knowledge, attitude, and the preventive practice towards food poisoning among postgraduate students in Universiti Putra Malaysia. These will provide information for interventional program if the level of knowledge, attitude, or preventive practice is discovered to be poor, and the factors associated tend to be unsatisfactory.

## Methods

### Study design

The study was a cross-sectional design to determine the knowledge, attitude, and preventive practice of food poisoning and its factors among postgraduate students in a public university in Selangor. The study setting was a tertiary learning institution known as Universiti Putra Malaysia. Universiti Putra Malaysia is a leading research university located in Serdang, a city in Selangor, Malaysia, and it is next to Malaysia’s administrative capital city, Putrajaya. As a world-famous center of learning and research, Universiti Putra Malaysia has attracted students and staffs from all around the globe, making the center a well-respected global entity. The participants’ recruitment period into the study and collection of the data was from 1^st^ July to 2^nd^ November 2020.

### Ethical consideration

The researcher acquired ethical approval for this study from the Faculty of Medicine and Health Sciences medical research and ethics committee, Universiti Putra Malaysia [Ethical approval reference number: UPM/TNCPI/RMC/JKEUPM1.4.18.2 (JKEUPM) 28-10-2019]. Participation by the participants who were able to give informed consent as subjects was voluntary and that they can withdraw at any time during the data collection. All potential subjects were sufficiently informed about the aims, procedures, no conflicts of interest for the research, the researcher’s institutional affiliation and background, expected benefits of this study, post-study benefits, and not generating any discomfort because all data are treated with confidentially and anonymity.

### Participants

Only those who are registered for postgraduate studies in Universiti Putra Malaysia from whole faculties and institutes on the main campus Serdang, willing to participate and provided consent were recruited for this study. The participants’ sources were from a list of registered students that the Universiti Putra Malaysia Graduate School Office provided. Simple random sampling was applied to select the participants.

### Variables

#### Outcomes

The outcomes for knowledge as a dependent variable are either poor or good knowledge. The outcomes of attitude as a dependent variable are either unacceptable or acceptable attitude. The outcomes of preventive practice as a dependent variable are either poor or good preventive practice.

#### Exposures

The exposures, predictors, effect modifiers, or potential confounders in this study are: awareness of food poisoning outbreak; source of the information about the food poisoning outbreak, if the participant says yes; the previous history of food poisoning illness; cause of the food poisoning illness, if the participant says yes; educational level; income; employment status; age; marital status; gender; and citizen.

#### Operational definition of outcomes

*Knowledge*. The definition of knowledge in this study is the level of the individual’s understanding of the causes of food poisoning, high-risk food, complication/effects of food poisoning, detecting spoiled food, and how to prevent food poisoning. The level of knowledge is divided into two categories, good knowledge and poor knowledge. Good knowledge shows from a score of 80% and above according to the section. And poor knowledge shows a score below 80% (79% and below) according to the section.

*Attitude*. The definition of attitude in this study concentrates on the attitude towards food contamination, food premise hygiene, cooked food temperature regulation, hand hygiene, unhygienic food handling, health-seeking behavior, and food safety. The level of attitude is divided into two categories, acceptable attitude and unacceptable attitude. Acceptable attitude shows a score from 80% and above according to the section. Unacceptable attitude shows a score below 80% (79% and below) according to the section.

*Preventive practice*. Preventive practice in this study is defined as the tradition which is habitually carried out (Hand hygiene, food safety, food premise hygiene, health-seeking behavior, personal hygiene, and food hygiene) to prevent food poisoning. The level of preventive practice is divided into two categories, good practice and poor practice. According to the section, good practice shows a score from 80% and above, and poor practice shows a score below 80% (79% and below).

#### Operational definition of exposures

*Gender*. The operational definition of gender is male and female.

*Age*. The operational definition of age is in two classes, less than 35 years old (< 35 years old) and 35 years old and above (≥ 35 years old).

*Citizen*. A citizen is defined as Malay, Chinese, Indian, or others. But for analysis, it is further categorized into Malaysian and non-Malaysian. Malaysian are Malay, Chinese, or Indians, while non-Malaysian, are the other respondents that took part in the study.

*Marital status*. Marital status is defined as either single, married, separated, divorced, or widowed. The operational definition is divided into two groups, single, separated, divorced, or widowed as one group and married as another group.

*Educational level*. Educational level is defined as the distribution of education from elementary education, primary school (6th grade), secondary education (5th grade), Certificate /STPM / A-level / GCE / Foundation /Matriculation / Diploma, to Tertiary research / (Degree / Master / PhD). For further analysis, the educational level is divided into the non-first-degree holder and first-degree holder.

*Employment status*. Employment status is defined as either self-employed, working in the public sector, working in the private sector, or not working. For further analysis, employment status will be categorized into working (self-employed, public sector, private sector) and not working.

*Average monthly income*. Monthly income is defined as the average monthly income in Malaysian Ringgit earned by the bottom 40% (B40). The average monthly income is 3,264 ringgits. For further analysis, the average monthly income is divided into those who earned below the average monthly income and those who earned equal to or above the average monthly income [28].

*Food poisoning outbreak*. The operational definition of food poisoning outbreak in this study is those that are not aware of food poisoning outbreak and those that are aware of food poisoning outbreak. The corresponding answer will be either ’no, I am not aware’ or ’yes, I am aware. And for those respondents that are aware who said yes, the question that follows is that ’what was the source of information on the food poisoning outbreak?’ according to the section.

*Food poisoning illness*. The operational definition of food poisoning illness in this study is those respondents who had a previous history of food poisoning illness (who said ’yes’) and those who had never had a previous history of food poisoning illness (who said ’no’). For those respondents that said yes, a question was asked ’What was the cause of their food poisoning illness?’ according to the section.

### Data sources/measurements

The sources of data for each of the variables of interest are postgraduate students in Universiti Putra Malaysia. And the method of assessment was a set of questionnaires on knowledge, attitude, and preventive practice. The instrument used was a validated knowledge, attitude, and practice questionnaire with a Cronbach Alpha reliability estimate of 70% [29]. The questionnaire had 73 questions categorized into four sections: sociodemographic factors, knowledge, attitude, and preventive practice concerning food poisoning.

The sociodemographic section comprised of 11 questions which consisted of respondents’ gender, age, citizen, marital status, educational level, employment status, average monthly income in Malaysian Ringgit, awareness of food poisoning outbreak, if yes what was the source of information of the outbreak, previous history of food poisoning illness, if yes, what was the cause of the food poisoning illness? Questions in this section were outlined to be typed written in the spaces provided on the questionnaire.

The knowledge section consisted of 42 questions grouped into five categories: respondents’ knowledge of causes of food poisoning, high-risk foods, complications/effects of food poisoning, how to detect spoiled food, and how to prevent food poisoning. Each question was planned to be answered in the right or wrong format. Negative knowledge statements were reversed.

The attitude section is comprised of 10 questions related to respondents’ attitudes concerning food poisoning prevention. The questions were designed to examine the respondent’s attitude towards the likelihood of foods being contaminated during food handling, reporting unhygienic food handling and food poisoning. Questions in this section were planned to be answered on a Likert scale (Strongly disagree, Disagree, Neutral, Agree, Strongly Agree).

The preventive practice items consist of ten questions concerning personal hygiene, food safety, food premise hygiene, reporting food poisoning, and food hygiene preventive practices. Questions in this section were created to be answered on a Likert scale (Never, Rarely, Sometimes, Always).

### Bias

In order to avoid being biased, simple random probability sampling was utilized. The study was designed to ensure that only the target population was included. The questionnaire was validated, and its reliability was checked in a prior study, and questions that may elicit favorable answers were reviewed and adjusted. Moreover, the response rate was reasonable.

### Study size

The estimation of the sample size was based on the research objectives for this study. The sample size estimation value was 208. An overall of 208 respondents was chosen for this study based on the calculation for the objectives. But anticipating a 10% non-response rate, the actual sample size that was used was 212 respondents.

To determine the level of knowledge, attitude, and preventive practices of food poisoning among postgraduate students, calculation for this study’s sample size was referred to the previous study by [9] where the values of knowledge, attitude, and practice were obtained: good knowledge: 89.36% (0.8936); poor attitude: 34.26% (0.3426); poor practice: 19.94% (0.1994). The formula for the calculation was referred to [30]:

n=Z21−α2P(1−P)d2 (1)

Where n = number of sample size; Z^2^ 1-α/_2_ = 1.96^2^; P = prevalence; d = 0.01

By applying the prevalence values that was referred to the prior study done by [9] (P = 0.8936 for knowledge; P = 0.3426 for attitude; P = 0.1994 for practice), the sample size required for the objectives were 36 (for knowledge), 86 (for attitude), 16 (for practice).

The calculation to determine associations between the respondents’ demographic and socioeconomic factors, awareness of food poisoning outbreak, source of information about the outbreak, previous history of food poisoning illness with knowledge, attitude, and the preventive practice of food poisoning among postgraduate students for this study was referred to the previous research by [31] to obtain the values of the percentage of exposed with the outcome *μT* = 68.8% and percentage of un-exposed with the outcome *μc* = 31.2%. While the formula for the calculation was referred to the prior study by [32].


$$n={\left[\frac{z\propto \sqrt{2uc(1-uc)}+z_{\beta }\sqrt{\left[u_{T}(1^{-\mu }T)+u_{c}(1-uc)\right]}}{u_{T}-uc}\right]}^{2}$$ (2)

Where n = sample size; μT = proportion of exposed with the outcome; μ_C_ = proportion of unexposed with the outcome. By applying the proportion values: μ_T_ = 0.688 (68.8%) and μ_C_ = 0.312 (31.2%), the sample size required for this objective was 70. Therefore, summing up the sample size estimations (138+70), the total was 208.

### Quantitative variables

Three questions in the sociodemographic section, "age", "what was the source of information on the food poisoning outbreak" and "what was the cause of the food poisoning illness" were quantitative variables whose values resulted from counting participants’ answers to the first, second, and third questions. However, for the first and third questions, it was subsequently categorized into those below and above 35 years of age, and either microbial or non-microbial causes, respectively. Because according to pieces of literature [1, 33, 34], the causes of food poisoning are either microbial (for example, *Staphylococcus aureus*) or non-microbial (For example, mercury food poisoning). So only those respondents who diagnosed the cause at a health center and stated the etiological agent of the food poisoning illness correctly were considered to have correctly detected the cause of their food poisoning illnesses.

### Statistical methods

Data were analyzed using the Statistical Package for Social Sciences version 27. A descriptive analysis that includes frequencies and percentages was performed. And also, descriptive analysis to test the relationships between the research variables. It involved crosstabulation that included chi-square tests and risk estimates. Binary logistic regression was applied for determining the predictors of knowledge and attitude towards food poisoning and for the control of confounding. No significant values were found between preventive practice and the sociodemographic factors; therefore, the binary logistic regression was not performed. No missing data were observed. The level of significance in this research was fixed up at p<0.05.

## Results

### Participants

Two hundred and twelve postgraduate students from Universiti Putra Malaysia appropriately answered the given questionnaire. And the response rate (208/212*100) was 98%.

### Description of the respondents based on demographic and socioeconomic characteristics

Of the 212 respondents that participated in this study, the majority were male (59.0%), less than 35 years of age (77.4%), non-Malaysian (52.8%), single (58.0%), first-degree holders (98.6%), not working (53.8%), and had an average monthly income less than RM3,264 (82.1%) **(Table 1)**.

**Table 1:** Distribution of the respondents based on demographic (gender, age, citizen) and socioeconomic (marital status, educational level, employment status, average monthly income) characteristics (N = 212).

Variable	Study group n = 212	
	N	(%)
**Gender**		
Male	125	59.0
Female	87	41.0
**Age**		
<35 years old	164	77.4
≥35 years old	48	22.6
**Citizen**		
Malaysian	100	47.2
Non-Malaysian	112	52.8
**Marital status**		
Single	123	58.0
Married	89	42.0
**Educational level**		
First-degree-holder	209	98.6
Non-first-degree holder	3	1.4
**Employment status**		
Working	98	46.2
Not working	114	53.8
**Average monthly income in Malaysian Ringgit (RM)**		
<3,264	174	82.1
≥3,264	38	17.9

### Description of the respondents based on the awareness of food poisoning outbreak

The analysis of the result of this study indicated that the majority of the respondents (67%) are aware of food poisoning outbreak, while a minority of them (33.0%) are not aware of food poisoning outbreak **(Table 2).**

**Table 2:** Distribution of the respondents (%) according to the awareness of food poisoning outbreak (N = 212).

Awareness item	n (%)
Have you ever heard about food poisoning outbreak?	
Yes	142 (67.0)
No	70 (33.0)

### Description of the respondents based on what was the source of the information of the food poisoning outbreak

Table 3 shows the distribution of the respondents according to their source of information of food poisoning outbreak. This study found that the majority of the respondents (67.0%) had heard about a food poisoning outbreak. And among the majority, 21.69% heard about food poisoning outbreak on television, 21.23% from the internet, 8.02% from a newspaper, 7.55% from Online journals, 3.30% from friends, 1.89% from Facebook, 0.5% from their community, 0.5% from their nurse, 0.5% after drinking raw milk for the second time, 0.5% were informed about it by his or her parents, 0.5% were informed about it by his or her relatives, 0.5% were informed about it in a restaurant, and 0.5% were informed about it on the radio.

**Table 3:** Distribution of the respondents (%) according to what was the source of information on the food poisoning outbreak (N = 212).

Source(s) of information	n (%)
No, I have not heard, so there is no information.	70 (33.0)
Television (news)	46 (21.69)
Newspaper	17 (8.02)
Online journals	16 (7.55)
Internet	45 (21.23)
Friends	7 (3.30)
Facebook	4 (1.89)
Community	1 (0.5)
Nurse	1 (0.5)
Second time drinking raw milk	1 (0.5)
From home, parent’s awareness	1 (0.5)
Relatives	1 (0.5)
Restaurant	1 (0.5)
Radio	1 (0.5)

### Description of the respondents based on the previous history of food poisoning illness

Table 4 shows the distribution of the respondents according to their previous history of food poisoning illness. The analysis of the data disclosed that the majority of the respondents (55.7%) have ever had a history of food poisoning illness, while a minority of them (44.3%) have never had a history of food poisoning illness.

**Table 4:** Distribution of respondents (%) according to the previous history of food poisoning illness.

Previous history of food poisoning illness item	n (%)
Have you ever had a history of food poisoning before?	
Yes	118 (55.7)
No	94 (44.5)

### Description of the respondents based on what was the cause of their food poisoning illness

Table 5 shows the distribution of respondents according to their causes of food poisoning illnesses. 2 (0.9%) of the respondents said that the cause of their food poisoning illness was salmonellosis. 1 (0.5%) of the respondents said that the cause of the respondent’s food poisoning illness was mushroom food poisoning.

**Table 5:** Distribution of the respondents according to what was the cause of their food poisoning illnesses (N = 212).

If yes, what was the cause of the food poisoning illness?	n (%)
No, I never had a history of food poisoning illness before.	94 (44.3)
Retail food restaurant	16 (7.5)
Expired food	10 (4.8)
Unhygienic food handling	10 (4.7)
Food	8 (3.8)
Chicken	7 (3.3)
Water pollution	6 (2.8)
Meat	6 (2.8)
Improperly cooked food	5 (2.4)
Food spoilage	5 (2.4)
Seafood	4 (1.9)
Street food vendor	3 (1.4)
Cross contamination	2 (0.9)
Microbes	2 (1.0)
Salmonellosis	2 (0.9)
Vegetable and poultry meat	1 (0.5)
Lack of personal hygiene	1 (0.5)
Milk	1 (0.5)
Keeping food for days without warming or covering it	1 (0.5)
Eggs	1 (0.5)
Foodborne pathogen	1 (0.5)
Expired cheese	1 (0.5)
Raw milk	1 (0.5)
Nasi lemak	1 (0.5)
Red onion	1 (0.5)
Heavy metals	1 (0.5)
Beans	1 (0.5)
Chinese milk scandal	1 (0.5)
Sausage	1 (0.5)
Insect borne food poisoning	1 (0.5)
Zoonosis	1 (0.5)
Fecal matter	1 (0.5)
Ice adulteration	1 (0.5)
Fruits and vegetables	1 (0.5)
Mushroom food poisoning	1 (0.5)
Inadequate food storage	1 (0.5)
Ground meat	1 (0.5)
Delivery food	1 (0.5)
Wet market supplied food	1 (0.5)
Open buffet	1 (0.5)
Contaminated cabbage and carrots with insecticide and rodenticide	1 (0.5)
Chemical runoff factories	1 (0.5)
Microorganisms, chemicals, and physical hazards	1 (0.5)
Fried hen	1 (0.5)
Catering service	1 (0.5)
Mayonnaise	1 (0.5)
Uncooked meat	1 (0.5)

### Description of the respondents based on knowledge of food poisoning

The result of the analysis of this study showed that the majority of the respondents had a poor level of knowledge (82.5%) of food poisoning, while a minority of the respondents (17.5%) had a good level of knowledge of food poisoning. The distribution of the respondents in conformity to each knowledge statement is indicated in Table 6. The result of the data analysis of this study revealed that of the four knowledge statements on what is the cause of food poisoning, the majority of the respondents answered three of the statements correctly. For the ten statements on which of the following is a high-risk food, this study found that the majority of the respondents answered only five of the items correctly. Of the fifteen statements on what are the effects/complications of food poisoning, a larger number of the respondents answered only seven of the statements correctly. Most of the respondents were able to answer the three statements on how to detect spoiled food correctly. A larger portion of the respondents answered the ten statements on how to prevent food poisoning correctly **(Table 6)**.

**Table 6:** Descriptive of knowledge statements of food poisoning among the respondents (N = 212).

No	Variable	Study group n = 212	
		Correct	Wrong
		n (%)	n (%)
	**What is the cause of food poisoning?**		
K1.	Bacteria	193 (91.0)	19 (9.0)
K2.	Virus	98 (46.2)	114 (53.8)
K3.	Parasites	111 (52.4)	101 (47.6)
K4.	Pesticide residues	121 (57.1)	91 (42.9)
	**Which of the following is a high-risk food?**		
K5.	Chicken	171 (80.7)	41 (19.3)
K6.	Meat	174 (82.1)	38 (17.9)
K7.	Bread	88 (41.5)	124 (58.5)
K8.	Dry food	136 (64.2)	76 (35.8)
K9.	Dairy product	162 (76.4)	50 (23.6)
K10.	Seafood	156 (73.6)	56 (26.4)
K11.	Rice	63 (29.7)	149 (70.3)
K12.	Food in non-dented can	46 (21.7)	166 (78.3)
K13.	Vegetables	99 (46.7)	113 (53.3)
K14.	Fruits	93 (43.9)	119 (56.1)
	**What are the effects/complications of food poisoning?**		
K15.	Diarrhea	196 (92.5)	16 (7.5)
K16.	Vomiting	196 (92.5)	16 (7.5)
K17.	Stomachache	202 (95.3)	10 (4.7)
K18.	Dryness of the lips	87 (41.0)	125 (59.0)
K19.	Lifeless or fatigue	102 (48.1)	110 (51.9)
K20.	Yellow eyes (jaundice)	35 (16.5)	177 (83.5)
K21.	Fever	140 (66.0)	72 (34.0)
K22.	Bloody feces	88 (41.5)	124 (58.5)
K23.	Muscle pain	95 (44.8)	117 (55.2)
K24.	Bleeding gums	98 (46.2)	114 (53.8)
K25.	Death	145 (68.5)	67 (31.6)
K26.	Kidney failure	92 (43.4)	120 (56.6)
K27.	Liver damage	76 (35.8)	136 (64.2)
K28.	Dehydration	174 (82.1)	38 (17.9)
K29.	Respiratory system failure	84 (39.6)	128 (60.4)
	**How do you detect spoiled food?**		
K30.	View physical changes in food	188 (88.7)	24 (11.3)
K31.	Spoiled food brings out bad smell	197 (92.9)	15 (7.1)
K32.	The change of taste in food	198 (93.4)	14 (6.6)
	**How can you prevent food poisoning?**		
K33.	Make sure foods are fully cooked.	201 (94.8)	11 (5.2)
K34.	I am using the same wiper cloth to wipe dishes and tables.	149 (70.3)	63 (29.7)
K35.	I am using the same cutting board to cut different raw foods.	141 (66.5)	71 (33.5)
K36.	Wash eggs thoroughly before cooking.	162 (76.4)	50 (23.6)
K37.	Wash hands with soap every time after using the toilet.	205 (96.7)	7 (3.3)
K38.	Use liquid soap when washing hands	178 (84.0)	34 (16.0)
K39.	Eat cooked food that is left at room temperature for 12–24 hours.	142 (67.0)	70 (33.0)
K40.	Separate raw food from cooked food.	174 (82.1)	38 (17.9)
K41.	Avoid the presence of animals and pests such as rats, flies, and cockroaches in food premises.	199 (93.9)	13 (6.1)
K42.	Practicing good personal hygiene	198 (93.4)	14 (6.6)

The association between demographic and socioeconomic factors with knowledge of food poisoning disclosed that the majority of the respondents had poor knowledge scores, and they were among those that are male (83.2%), 35 years old and above (87.5%), non-Malaysian (88.4%), married (88.8%), non-first-degree holders (100.0%), not working (83.3%), had an average monthly income greater than or equal to RM3264 (84.2%), were not aware of food poisoning outbreak (91.4%), and had no previous history of food poisoning illness (89.4%) **(Table 7)**. The bivariate analysis result displayed a significant association between citizen and poor knowledge of food poisoning (p = 0.018). The citizen that is non-Malaysian is 0.860 more likely to have poor knowledge compared to the citizen that is Malaysian with poor knowledge (Confidence Interval 95%: 0.756–0.978) **(Table 7)**.

**Table 7:** Association between demographic and socioeconomic factors, awareness of food poisoning outbreak, and previous history of food poisoning illness with the knowledge of food poisoning (N = 212).

Variable	Knowledge		P-value	Prevalence Ratio	95% CI	
	Poor	Good			Lower	Upper
	n (%)	n (%)				
**Gender**						
Male	104 (83.2)	21 (16.8)	0.764^a^	1.019	0.898	1.158
Female	71 (81.6)	16 (18.4)				
**Age**						
<35 years old	133 (81.1)	31 (18.9)	0.304^a^	0.927	0.814	1.055
≥35 years old	42 (87.5)	6 (12.5)				
**Citizen**						
Malaysian	76 (76.0)	24 (24.0)	0.018*	0.860	0.756	0.978
Non-Malaysian	99 (88.4)	13 (11.6)				
**Marital status**						
Single	96 (78.0)	27 (22.0)	0.042*	0.879	0.780	0.991
Married	79 (88.8)	10 (11.2)				
**Educational level**						
First-degree-holder	172 (82.3)	37 (17.7)	1.00^b^	0.823	0.773	0.876
Non-first-degree holder	3 (100.0)	0 (0.0)				
**Employment status**						
Working	80 (81.6)	18 (18.4)	0.745^a^	0.980	0.865	1.110
Not working	95 (83.3)	19 (16.7)				
**Awareness of food poisoning outbreak?**						
No	61 (91.4)	6 (8.6)	0.017*	1.170	1.045	1.309
Yes	111 (78.2)	31 (21.8)				
**Previous history of food poisoning illness?**						
No	84 (89.4)	10 (10.6)	0.020*	1.159	1.027	1.307
Yes	91 (77.1)	27 (22.9)				
**Average monthly income in RM.**						
<3264	143 (82.2)	31 (17.8)	0.766^a^	0.976	0.837	1.139
≥3264	32 (84.2)	6 (15.8)				

^a^ Chi-square test

* Significant by chi-square test.

The bivariate analysis result showed that there is a significant association between marital status and poor knowledge score of food poisoning (p = 0.042). The marital status married is 0.879 more likely to have poor knowledge compared to those that are single with poor knowledge of food poisoning (Confidence Interval 95%: 0.780–0.991) **(Table 7)**.

The bivariate analysis indicated that there is a significant association between awareness of food poisoning outbreak and poor knowledge score of food poisoning (p = 0.017). Those respondents that are not aware of food poisoning outbreak are 1.170 more likely to have poor knowledge of food poisoning compared to those that are aware with poor knowledge of food poisoning (Confidence Interval 95%: 1.045–1.309) **(Table 7)**.

The bivariate analysis indicated that there is a significant association between the previous history of food poisoning illness and poor knowledge score of food poisoning (p = 0.020). Those who had no previous history of food poisoning illness are 1.159 more likely to have poor knowledge compared to those who had a previous history of food poisoning illness with poor knowledge of food poisoning (Confidence Interval 95%: 1.027–1.307) **(Table 7)**.

This study did not find any significant association between the respondent’s gender, age, educational level, employment status, and average monthly income in Malaysian Ringgit with the knowledge of food poisoning **(Table 7)**.

The result of the analysis of the association of the respondents’ knowledge and attitude towards food poisoning showed that the majority of the respondents had acceptable attitude towards food poisoning (81.1%), and they are among those with good knowledge of food poisoning. But no significant association was found between knowledge with attitude towards food poisoning **(Table 8)**, and also with the preventive practice of food poisoning **(Table 9)**.

**Table 8:** Association between knowledge with attitude towards food poisoning among the respondents.

Variable	(n = 212)		P-value	Prevalence Ratio	95% CI	
	Attitude					
	Unacceptable	Acceptable			Lower	Upper
	n (%)	n (%)				
**Knowledge**						
**Poor (score< 80)**	59 (33.7)	116 (66.3)	0.08^a^	0.561	0.279	1.129
**Good (score≥ 80)**	7 (18.9)	30 (81.1)				

a Chi-square test.

**Table 9:** Association between knowledge with the preventive practice of food poisoning among the respondents.

Variable	(n = 212)		p-value	Prevalence Ratio	95% CI	
	Preventive practice					
	Poor n (%)	Good n (%)			Lower	Upper
**Knowledge**			0.109^a^			
**Poor (score<**	82 (46.9)	93		0.692	0.424	1.131
**80)**		(53.1)				
**Good**	12 (32.4)	25				
**(score≥ 80)**		(67.6)				

^a^ Chi-square test.

Binary logistic regression analysis was conducted to recognize the effects of independent variables (citizen, marital status, awareness about food poisoning outbreak, and previous history of food poisoning illness) on knowledge of food poisoning among the respondents. Of the four predictor variables, only three were statistically significant: marital status, awareness of food poisoning outbreak, and previous history of food poisoning illness. The result indicated that there was a weak association (R^2^ = 0.114) between prediction and grouping. And the model explained 11.4% (Nagelkerke R^2^) of the variance in knowledge **(Table 10)**.

**Table 10:** Predictors influencing knowledge of respondents towards food poisoning.

Variable	Logistic Coefficient(B)	SE	Adjusted Odd Ratio	95% CI		*p-*value
				Lower	Upper	
**Marital status**						
Single			1			
Married	0.851	0.409	2.342	1.051	5.220	0.037*
**Awareness of food poisoning outbreak?**						
No			1			
Yes	-1.006	0.483	0.366	0.142	0.942	0.037*
**Previous history of food poisoning illness?**						
No			1			
Yes	-0.809	0.410	0.445	0.199	0.996	0.049*
Constant	2.507	0.505	12.266			0.000
X^2^	15.168					0.002
Df	3					

*Significant p<0.05.

Method = Backward LR.

R^2^ = 0.114, overall percentage = 82.5%.

The result of the analysis showed that respondents who are married are 2.342 times more likely to exhibit good knowledge of food poisoning than the respondents that are single. Those respondents who are aware of food poisoning outbreak are 0.366 times less likely to exhibit good knowledge of food poisoning than those respondents who are not aware of food poisoning outbreak. And also, those respondents who had a previous history of food poisoning illness are 0.445 times less likely to exhibit good knowledge of food poisoning than those who had no previous history of food poisoning illness **(Table 10).**

### Description of the respondents based on attitude towards food poisoning

The result of the analysis indicated that for the nine out of the ten statements on attitude towards food poisoning, most of the respondents answered strongly agreed with each of the items. But, concerning the statement, "I care if I see food handlers smoking during food preparation and handling," the majority of them answered neither agreed nor disagreed **(Table 11)**.

**Table 11:** Descriptive of attitude statements of food poisoning among the respondents (N = 212).

No	Variable	Study group				
		n = 212				
		Strongly Disagree	Disagree	Neutral	Agree	Strongly agree
		n (%)	n (%)	n (%)	n (%)	n (%)
A1	I would choose a restaurant where food handlers wear gloves when handling food.	18 (8.5)	6 (2.8)	22 (10.4)	58 (27.4)	108 (50.9)
A2	I would not choose a restaurant where the chef has long nails.	32 (15.1)	4 (1.9)	17 (8.0)	47 (22.2)	112 (52.8)
A3	I will make sure of the cleanliness grade of the premises when choosing eateries.	18 (8.5)	1 (0.5)	12 (5.7)	59 (27.8)	122 (57.5)
A4	I will not buy cooked foods that are exposed to room temperature for a long time.	17 (8.0)	12 (5.7)	21 (9.9)	54 (25.5)	108 (50.9)
A5	I will make sure the eateries I visit are clean.	15 (7.1)	6 (2.8)	11 (5.2)	51 (24.1)	129 (60.8)
A6	I will make sure to always wash my hands with soap before eating.	17 (8.0)	7 (3.3)	15 (7.1)	47 (22.2)	126 (59.4)
A7	I will be reporting to the authorities (such as local authorities) in the event of operating activities and the provision of unhygienic foods in food premises.	13 (6.1)	20 (9.4)	59 (27.8)	48 (22.6)	72 (34.0)
A8	I will report to the authorities (such as local health authorities) about food poisoning.	13 (6.1)	16 (7.5)	45 (21.2)	56 (26.4)	82 (38.7)
A9	I need to see a doctor if I have any signs of food poisoning.	17 (8.0)	8 (3.8)	22 (10.4)	53 (25.0)	112 (52.8)
A10	I care if I see food handlers smoking during food preparation and handling.	13 (6.1)	6 (2.8)	85 (40.1)	38 (17.9)	70 (33.0)

The result of the analysis of this study for the association of the respondents’ attitude with demographic and socioeconomic factors showed that the majority of the respondents had acceptable attitude scores towards food poisoning, and they are among those that are female (79.3%), less than 35 years old (69.5%), Malaysian (70.0%), married (73.0%), first-degree holder (68.9%), working (69.4%), had an average monthly income less than 3264 MYR (69.0%), are aware of food poisoning outbreak (74.6%), and had a previous history of food poisoning illness (72.9%) **(Table 12)**.

**Table 12:** Association of demographic and socioeconomic factors, awareness of food poisoning outbreak, and previous history of food poisoning illness with attitude towards food poisoning among the respondents (N = 212).

Variable	Attitude		P-value	Prevalence Ratio	95% CI	
	Unacceptable	Acceptable			Lower	Upper
	n (%)	n (%)				
**Gender**						
Male	48 (38.4)	77 (61.6)	0.006*	1.856	1.163	2.962
Female	18 (20.7)	69 (79.3)				
**Age**						
<35 years old	50 (30.5)	114 (69.5)	0.708^a^	0.915	0.576	1.452
≥35 years old	16 (33.3)	32 (66.7)				
**Citizen**						
Malaysian	30 (30.0)	70 (70.0)	0.737^a^	0.933	0.624	1.396
Non-Malaysian	36 (32.1)	76 (67.9)				
**Marital status**						
Single	42 (34.1)	81 (65.9)	0.265^a^	1.266	0.831	1.929
Married	24 (27.0)	65 (73.0)				
**Educational level**						
First-degree holder	65 (31.1)	144 (68.9)	1.000^b^	0.933	0.186	4.682
Non-first- degree- holder	1 (33.3)	2 (66.7)				
**Employment status**						
Working	30 (30.6)	68 (69.4)	0.880^a^	0.969	0.648	1.450
Not working	36 (31.6)	78 (68.4)				
**Average monthly income in RM.**						
<3264	54 (31.0)	120 (69.0)	0.948^a^	0.983	0.586	1.649
≥3264	12 (31.6)	26 (68.4)				
**Awareness of food poisoning outbreak?**						
No	30 (42.9)	40 (57.1)	0.010*	1.690	1.143	2.499
Yes	36 (25.4)	106 (74.6)				
**Previous history of food poisoning illness?**						
No	34 (36.2)	60 (63.8)	0.157^a^	1.334	0.894	1.989
Yes	32 (27.1)	86 (72.9)				

^a^ Chi-square test

* Significant by chi-square test.

The bivariate analysis result showed a significant association between gender and unacceptable attitude towards food poisoning (p = 0.006). The male gender is 1.856 more likely to have an unacceptable attitude compared to the female gender with an unacceptable attitude towards food poisoning (Confidence Interval 95%: 1.163–2.962) **(Table 12)**.

The bivariate analysis revealed a significant association between awareness of food poisoning outbreak and unacceptable attitude towards food poisoning (p = 0.010). Those who had no awareness of food poisoning outbreak are 1.690 more likely to have an unacceptable attitude compared to those who are aware of food poisoning outbreak with unacceptable attitude towards food poisoning (Confidence Interval 95%: 1.143–2.499) **(Table 12)**.

The result of the analysis of the association of the respondents’ attitude with preventive practice of food poisoning showed that the majority of the respondents had good preventive practice (77.1%), and they are among those with an acceptable attitude towards food poisoning. The bivariate analysis indicated that there is a significant association between attitude and preventive practice of food poisoning (p = 0.004). Those respondents with unacceptable attitude are 0.551 times more likely to have poor preventive practice compared to those respondents who had acceptable attitude with poor preventive practice (Confidence Interval 95%: 0.366–0.830) **(Table 13).**

**Table 13:** Association between the respondents’ attitude with the preventive practice of food poisoning.

Variable	(n = 212)		P-value	Prevalence Ratio	95% CI	
	Preventive practice					
	Poor n (%)	Good n (%)			Lower	Upper
**Attitude:**						
Unacceptable	39 (41.5)	55	0.004*	0.551	0.366	0.830
(score<80)		(58.5)				
Acceptable	27 (22.9)	91				
(score≥80)		(77.1)				

^a^ Chi-square test

* Significant by chi-square test.

Binary logistic regression analysis was conducted to recognize the effects of independent variables (gender and awareness of food poisoning outbreak) on attitude towards food poisoning among the respondents. The two predictor variables were statistically significant: gender and awareness of food poisoning outbreak. The result indicated that there was a weak association (R^2^ = 0.088) between prediction and grouping. And the model explained 8.8% (Nagelkerke R^2^) of the variance in attitude. The result of the analysis showed that respondents who are of the female gender are 0.426 times less likely to exhibit acceptable attitude towards food poisoning than the male respondents. Respondents who are aware of food poisoning outbreak are 0.462 times less likely to exhibit acceptable attitude towards food poisoning than those respondents who are not aware of food poisoning outbreak **(Table 14).**

**Table 14:** Predictors influencing the attitude of respondents towards food poisoning.

Variables	Logistic Coefficient (B)	SE	Adjusted Odd Ratio	95% CI		*P*-value
				Lower	Upper	
**Gender**						
Male			1			
Female	-0.853	0.327	0.426	0.225	0.808	0.009*
**Awareness of food poisoning outbreak?**						
No			1			
Yes	-0.772	0.315	0.462	0.249	0.856	0.014*
**Constant**	0.014	0.269	1.014			0.958
**X** ^**2**^	13.739					0.001
**Df**	2					

*Significant P<0.05.

Method = Backward LR.

R^2^ = 0.088, overall percentage = 68.9%.

### Description of respondents based on the preventive practice of food poisoning

The analysis results regarding the preventive practices of food poisoning indicated that out of the ten statements in this section, the majority of the respondents answered ’Always’ for eight of the statements. But the majority of them answered ’Sometimes’ for two of the statements in this section **(Table 15)**.

**Table 15:** Descriptive of preventive practice statements of food poisoning among respondents.

No	Variable	Study group			
		n = 212			
		Never	Rarely	Sometimes	Always
		n (%)	n (%)	n (%)	n (%)
P1.	I wash my hands until clean before eating.	4 (1.9)	5 (2.4)	34 (16.0)	169 (79.7)
P2.	I use liquid soap instead of bar soap when washing my hands.	5 (2.4)	11 (5.2)	82 (38.7)	114 (53.8)
P3.	I will reject eateries where food handlers smoke when handling food.	11 (5.2)	22 (10.4)	75 (35.4)	104 (49.1)
P4.	I will check the hygiene grade of the premises before entering the eatery.	5 (2.4)	21 (9.9)	76 (35.8)	110 (51.9)
P5.	I will see a doctor if there are symptoms of food poisoning.	4 (1.9)	24 (11.3)	70 (33.0)	114 (53.8)
P6.	I will reject a restaurant where food handlers do not wear gloves when handling food.	11 (5.2)	44 (20.8)	88 (41.5)	69 (32.5)
P7.	I will not choose a restaurant where food handlers do not wear gloves when handling food.	13 (6.1)	40 (18.9)	78 (36.8)	81 (38.2)
P8.	I will reject a restaurant where food handlers do not wear head coverings.	14 (6.6)	57 (26.9)	83 (39.2)	58 (27.4)
P9.	I will smell a food first to make sure it is not spoiled.	10 (4.7)	40 (18.9)	69 (32.5)	93 (43.9)
P10.	I do not spit around eateries.	84 (39.6)	9 (4.2)	26 (12.3)	93 (43.9)

This study’s result analysis for the association between preventive practice with the sociodemographic factors disclosed that the majority of the respondents had a good preventive practice of food poisoning. They were among those that were female (59.8%), greater than or equal to 35 years old (64.6%), Malaysian (59.0%), married (58.4%), non-first-degree holder (66.7%), not working (56.1%), with average monthly income greater than or equal to RM3264 (65.8%), aware of food poisoning outbreak (57.7%), and had no previous history of food poisoning illness (58.5%) **(Table 16)**. This study did not find any significant association between the preventive practice of food poisoning with all the demographic and socioeconomic variables, awareness of food poisoning outbreak, and the previous history of food poisoning illness among the respondents **(Table 16)**.

**Table 16:** Association between demographic and socioeconomic factors, awareness of food poisoning outbreak, and the previous history of food poisoning illness with the preventive practice of food poisoning among the respondents (n = 212).

Variable	Preventive practice		P- value	Prevalence Ratio	95% CI	
	Poor	Good			Lower	Upper
	n (%)	n (%)				
**Gender**						
Male	59 (47.2)	66 (52.8)	0.315^a^	1.173	0.855	1.610
Female	35 (40.2)	52 (59.8)				
**Age**						
< 35 years old	77 (47.0)	87 (53.0)	0.157^a^	1.326	0.875	2.008
≥ 35 years old	17 (35.4)	31 (64.6)				
**Citizen**						
Malaysian	41 (41.0)	59 (59.0)	0.355^a^	0.866	0.638	1.176
Non-Malaysian	53 (47.3)	59 (52.7)				
**Marital status**						
Single	57 (46.3)	66 (53.7)	0.490^a^	1.115	0.817	1.522
Married	37 (41.6)	52 (58.4)				
**Educational level**						
First degree holder	93 (44.5)	116 (55.5)	1.000^b^	1.335	0.268	6.661
Non-first-degree holder	1 (33.3)	2 (66.7)				
**Employment status**						
Working	44 (44.9)	54 (55.1)	0.879^a^	1.024	0.757	1.385
Not working	50 (43.9)	64 (56.1)				
**Average monthly income in RM.**						
<3264	81 (46.6)	93 (53.4)	0.165^a^	1.361	0.852	2.175
≥3264	13 (34.2)	25 (65.8)				
**Awareness of food poisoning outbreak?**						
No	34 (48.6)	36 (51.4)	0.384^a^	1.150	0.845	1.565
Yes	60 (42.3)	82 (57.7)				
**Previous history of food poisoning illness?**						
No	39 (41.5)	55 (58.5)	0.456^a^	0.890	0.654	1.211
Yes	55 (46.6)	63 (53.4)				

No significant association was observed between preventive practice and knowledge of food poisoning among the respondents **(Table 9)**. However, a significant association was found between preventive practice with attitude towards food poisoning among the respondents **(Table 13)**. There was no significant association between preventive practice and the demographic and socioeconomic factors; consequently, no binary logistic regression was performed.

## Discussion

### Key results

#### Demographic and socioeconomic factors

This study discovered that the majority of the respondents who participated were male, less than 35 years of age, non-Malaysian, single, first-degree holders, not working, and had an average monthly income of less than RM3264. The previous study that was done by [35, 36] noticed that the majority of the respondents in their study who participated were male, which conforms to this study’s finding. [37] in their study also noted that the larger part of the respondents were aged less than 35 years.

A greater number of the respondents in this study who took part were non-Malaysian; this is similar to the prior finding in the study by [38]. This study found that the majority of the respondents who took part were single; this is consistent with what was found previously in the studies by [39–41]. In addition, a larger percentage were first-degree holders; this corresponds to the earlier findings by [42, 43]. The finding of this study noted that most of the respondents were not working; this agrees with the previous discovery by [44]. Also, a greater number had an average monthly income of less than RM3264, and this corresponds to the prior findings by [9, 45].

The majority of the respondents in this study were aware of food poisoning outbreak, and the source of their information was television, the internet, newspaper, Online journals, friends, Facebook, community, nurse, drinking raw milk for the second time, information from their parents, relatives, restaurant, and radio. More than half of the respondents had a previous history of food poisoning illness. But, most of them that had a previous history of food poisoning illness did not correctly detect or confirm the causes of their food poisoning illness.

This study’s finding that most of the respondents are aware of food poisoning outbreak agrees with the earlier results in the studies by [9, 41], although the sample size of their study was less as compared to this study. Also, some findings of the source of information of the food poisoning outbreak are similar to the earlier studies that were done by [46–49].

This study found that a larger portion of the respondents had a previous history of food poisoning illness because they did not know how to prevent it. It implies that this portion of respondents had poor safe and hygienic food handling knowledge. Moreover, they had suffered food poisoning due to their lack of Healthcare Seeking Behavior that can contribute to their hygienic and safe food handling knowledge that will influence and motivate their attitude and subsequently manifest in their food handling practice. This study’s finding of a larger percentage who had a previous history of food poisoning illness is consistent with the earlier study that was done by [50]. This study noticed that a greater number of those who had a previous history of food poisoning illness did not correctly detect or confirm the microbial or non-microbial causes. No previous similar or contrary findings have been reported.

### Knowledge of food poisoning

The prevalence of postgraduate students (82.5%) in Universiti Putra Malaysia with poor knowledge of food poisoning was discovered to be high. The majority of the respondents in this study had a poor understanding of the domains of a virus as a microbial cause of food poisoning, low-risk foods like bread, food in the non-dented can, and high-risk foods like rice, fruits, and vegetables. These are crucial areas in examining the interviewees’ understanding of the causes of food poisoning. Because if they have good knowledge, they will be able to associate it with consuming properly cooked food, practicing good personal hygiene, preventing the presence of animals and pests in food premises, separating raw food from cooked food, and other processes that can lead to food contamination.

They had a poor understanding of fatigue, jaundice, bloody feces, muscle pain, dryness of the lips, kidney failure, liver damage, and respiratory system failure as a complication or effect of food poisoning and bleeding gums as not a complication of food poisoning. A greater number had a poor understanding of these areas and could not link these domains to the causes, high risks foods of, and how to prevent food poisoning. This study’s finding that the majority of the respondents are having poor knowledge of food poisoning is similar to the earlier discoveries by [51, 52]. However, it is contrary to the earlier findings by [9–11].

### Attitude towards food poisoning

The proportion of postgraduate students (68.9%) in Universiti Putra Malaysia with an acceptable attitude towards food poisoning was found to be high. This indicates that the majority of the postgraduate students had positive beliefs or opinions towards hygienic and safe food handling. But there are few respondents with negative beliefs or opinions regarding hygienic and safe food handling.

A larger portion of the respondents neither agreed nor disagreed with the statement "They care if they see food handlers smoking during food preparation and handling." It indicates that the interviewees had negative beliefs or opinions concerning hygienic and safe food handling in this domain, putting them at risk of food poisoning. There is a break in continuity in this area because the result of this study revealed that they had a good knowledge score of relating practicing good personal hygiene as a preventive factor of food poisoning. When an individual smokes when handling food, the ashes, cigarette butts, and smoke can contaminate the food. Also, harmful bacteria can pass from the individual’s mouth to the individual’s hands and then to the food.

This study found that the majority of the respondents strongly agreed with the statements "They would choose a restaurant where food handlers wear gloves when handling food," "Would not choose a restaurant where the chef has long nails," "Will make sure of the cleanliness grade of the premises when choosing eateries," "Will make sure the eateries they visit is clean", and "Will make sure always to wash their hands with soap before eating". It indicates that the respondents have positive beliefs or opinions about hand hygiene and correct food premise cleanliness and sanitation which prevents microbial or non-microbial food contamination that can bring about food poisoning.

A larger number of the respondents strongly agreed with the statement, "They will not buy cooked foods that are exposed to room temperature for a long time." It shows that the interviewees have positive beliefs or opinions concerning the correct temperature handling of cooked food. It is important that ready-to-eat foods and particularly potentially risky foods are stored outside the 5⸰C to 60⸰C temperature range (danger zone) to lower the possibility of pathogenic microbial contamination and growth [53].

The majority of the respondents strongly agreed with the statements "They will be reporting to authorities (such as local authorities) in the event of operating activities and the provision of unhygienic foods in food premises" and "They will report to the authorities (such as local health authorities) about food poisoning." It reveals that the interviewees have positive beliefs or opinions about sustaining hygienic food preparation and reporting food poisoning to prevent the disease. A larger number of the respondents strongly agreed with the statement "They need to see a doctor if they have any signs of food poisoning." It shows that the interviewees have positive beliefs or opinions of preventing food poisoning through treatment-seeking behavior. They will be engaging in the action of dealing with food poisoning illness, which is a deviation from the state of good health, after perceiving themselves to have any symptoms of food poisoning. They have a high level of motivation to prevent food poisoning because they understand the positive benefits of treatment-seeking to nullify the perceived threat. This study’s finding is similar to the earlier studies that were done by [48, 52].

### The preventive practice of food poisoning

The percentage of postgraduate students in Universiti Putra Malaysia with good preventive practice was 55.7%, showing that more than half of the respondents had good preventive practice concerning food poisoning. But there are few remainders with poor preventive practices towards food poisoning.

The majority of the respondents sometimes "Will reject a restaurant where food handlers do not wear gloves when handling food," and "Will reject a restaurant where food handlers do not wear head coverings." It revealed a break in continuity in this area because the result of this study indicated that the respondents had a positive attitude of relating good personal hygiene as a preventive factor concerning food poisoning. But their preventive practice is communicated differently. They had poor traditions or habits of wearing gloves and head coverings. Head coverings and hand gloves act as a protective barrier against microbial and non-microbial causes of food poisoning that can contaminate food.

A larger percentage of the respondents had a good preventive practice regarding food poisoning by always "Washing their hands until clean before eating," "Not spitting around the eatery," and "Using liquid soap instead of bar soap when washing their hands." The interviewees had continuous good traditions or habits of hand hygiene and preventing food premise contamination that can result in food poisoning. Their knowledge in the area of practicing good personal hygiene and attitude towards hand hygiene, as well as food premises cleanliness, have contributed to their good preventive practices.

The majority of the respondents will always "Reject eateries where food handlers smoke when handling food." It shows that the interviewees have constant good traditions or habits in this area. Although, they had a negative attitude in this area which did not contribute to this good preventive practice. The majority of the respondents will always "Check the hygiene grade of the premise before entering the eatery," "Not choose a restaurant where food handlers do not wear gloves when handling food," and "Smell a food first to make sure it is not spoiled." It showed that the interviewees had constant good traditions or habits of preventing food and premise contamination that could lead to food poisoning. Their good knowledge in the domain of practicing good personal hygiene and how to detect spoiled food and the positive attitude towards correct food premise cleanliness and hygiene have contributed to their good preventive practices. A greater percentage of the respondents always “Will see a doctor if there are symptoms of food poisoning. The respondents’ good knowledge in some of the few domains of the complications of food poisoning because they had poor scores on the majority of the statements, and positive attitudes in the section of the need to see a doctor if they have any signs of food poisoning have promoted this unfailing good habit of treatment-seeking behavior.

### Factors associated with knowledge, attitude, and the preventive practice of food poisoning

Non-Malaysian respondents (88.4%) constituted a significantly higher percentage of respondents with poor knowledge of food poisoning as compared to Malaysian respondents (p<0.05). Non-Malaysian respondents had a poor understanding of viral causes of food poisoning, high-risk foods, and complications of food poisoning; therefore, they are at risk of contracting food poisoning. No similar or contrary earlier findings were found. However, the earlier study that was done by [54] discovered a significant association between gender and the level of knowledge. [43] in their study found that there was a significant association between age, gender, the field of study, and year of study with knowledge of food safety and hygiene among the respondents. While [55] in their study noticed that knowledge level was significantly associated with the level of formal education.

Respondents who are married (88.8%) represented a significantly higher proportion of respondents with poor knowledge of food poisoning as compared to single respondents (p<0.05). This poor understanding is likely because they do not understand the possibility of food risks that can evolve from high-risk foods, viral causes, and effects of food poisoning, which puts the married respondents at risk of food poisoning. This study’s finding is contrary to the prior finding by [51].

Respondents who were not aware of food poisoning outbreak (91.4%) constituted a higher percentage of respondents with poor knowledge of food poisoning as compared with those who are aware of food poisoning outbreak (p<0.05). Not being aware will escalate their risks of developing food poisoning since they are not knowledgeable of viral causes, high-risk foods, and repercussions of food poisoning that will enable them to recognize and intervene in their risk of contracting food poisoning. No similar or contrary previous studies were found.

Respondents with no previous history of food poisoning illness (89.4%) represented a significantly higher portion of respondents with poor knowledge of food poisoning (p<0.05). A greater percentage of the respondents who had no previous history of food poisoning illness had a poor understanding of food poisoning, which is similar to the findings of a prior study [49]. They have not suffered food poisoning illness before, and they had poor knowledge of viral causes, high-risk foods, and complications of food poisoning; this will predispose them to the risks of food poisoning.

Female respondents constituted a significantly higher percentage of respondents (79.3%) with acceptable attitude towards food poisoning as compared to the male respondents (p<0.05). The female respondents had acceptable beliefs or opinions on hand hygiene, food premise cleanliness, food contamination, food hygiene and safety, and also reporting food poisoning.

Respondents who are aware of food poisoning outbreak represented a significantly higher portion of respondents (74.6%) with acceptable attitude towards food poisoning as compared to respondents who are not aware with acceptable attitude (p<0.05). Respondents who recognized an incident in which two or more individuals experienced a similar illness resulting from consuming contaminated food constituted a significantly higher percentage of respondents with acceptable beliefs or opinions towards food poisoning. Likely the awareness had contributed to their acceptable beliefs; therefore, they are less likely to contract food poisoning.

Attitude towards food poisoning was significantly associated with preventive practice about food poisoning. Respondents who had an acceptable attitude towards food poisoning constituted a significantly higher percentage of the respondents with good preventive practice towards food poisoning (p<0.05). The acceptable beliefs or opinions regarding food poisoning have promoted their preventive practice concerning food poisoning.

Binary logistic regression indicated that married respondents are 2.342 times more likely to demonstrate good understanding of food poisoning as compared to the respondents that are single. Married interviewees in this study are a protective factor to good knowledge of food poisoning. As a result, it prevents the likelihood of food poisoning. A similar finding was found in the study performed by [56], although the association was not significant.

Those interviewees who are aware of food poisoning outbreak are 0.366 times less likely to demonstrate good understanding of food poisoning than those who are not aware of food poisoning outbreak. Being conscious of food poisoning outbreak in this study is a risk factor to the good knowledge of food poisoning. No similar or contrary previous studies were found. However, the study that was performed by [57] disclosed that educational level was a predictor of the knowledge of foodborne diseases.

Interviewees who had a previous history of food poisoning illness are 0.445 times less likely to exhibit good understanding of food poisoning than those who had no previous history of food poisoning illness. Respondents who had a previous history of food poisoning illness in this study are risk factors to the good knowledge of food poisoning. No similar or contrary previous studies were found.

The female gender is 0.426 times less likely to demonstrate an acceptable attitude concerning food poisoning. The female respondents are risk factors to acceptable attitude towards food poisoning, and therefore it increases the chances of the female gender developing food poisoning. No similar or contrary previous studies were found.

Respondents who are aware of food poisoning outbreak are 0.462 times less likely to demonstrate an acceptable attitude regarding food poisoning than those who are not aware with an acceptable attitude. Awareness of a food poisoning outbreak is a risk factor for exhibiting an acceptable attitude towards food poisoning because it increases the chance of the respondents to develop food poisoning. No similar or contrary prior studies were found. However, the study that was performed by [58] discovered that education significantly influenced the positive attitudes of the respondents regarding food safety.

## Limitations

The limitation of this study is that postgraduate students on the Bintulu campus located in Bintulu, Sarawak were not recruited into this study.

## Interpretation

Based on the evidence from this study, it can be inferred that the knowledge of food poisoning among postgraduate students in Universiti Putra Malaysia is poor. At the same time, the attitude and preventive practice towards the disease was acceptable and good, respectively. Citizen, marital status, awareness of food poisoning outbreak, and previous history of food poisoning illness of the respondents were significantly associated with the knowledge of food poisoning. Postgraduate students who are non-Malaysians, married, not aware of poisoning outbreak, and had no previous history of food poisoning illness were determined to have been influenced by the poor knowledge of food poisoning.

Gender and awareness of food poisoning outbreak of the respondents were significantly associated with attitude towards food poisoning. Being male and not aware of food poisoning outbreak was determined to influence the unacceptable attitude of postgraduate students in Universiti Putra Malaysia concerning food poisoning. Respondents’ attitude towards food poisoning was significantly associated with the preventive practice towards food poisoning. Those postgraduate students with the poor preventive practice were found to have an unacceptable attitude towards food poisoning. The attitude towards food poisoning was determined to have influenced the preventive practice of food poisoning among them.

Binary logistic regression disclosed that marital status, awareness of food poisoning outbreak, and previous history of food poisoning illness of the respondents are predictors of good knowledge of food poisoning. Postgraduate students who have married were not a confounding factor; they were a protective factor to the good knowledge of food poisoning. Postgraduate students who are aware of food poisoning outbreak and had a previous history of food poisoning illness were not confounding factors; they were risk factors to the good knowledge of food poisoning. Gender and awareness of food poisoning outbreak of the respondents are predictors of acceptable attitude towards food poisoning. Postgraduate students who are female and aware of food poisoning outbreak were not confounding factors. They were risk factors to the acceptable attitude towards food poisoning. Confirmation of the identified poor level of knowledge and factors affecting the level of knowledge, attitude, and preventive practice gives the required information on the reference yardsticks towards the risk of food poisoning among the postgraduate students. A suitable interventional program is highly proposed to prevent the potential risk of food poisoning outbreak among them.

## Generalizability of this study

A probability sample selected by simple random sampling is a sample in which every individual in the target population has the same chance of being included. It is a necessary condition for being able to generalize the findings. This cross-sectional study result was found to be effective among the interviewees who are postgraduate students in Universiti Putra Malaysia; therefore, the external validity or generalizability of this study result is not limited. And the conclusions of this study can be applied to other populations.

## Supporting information

S1 Appendix STROBE checklist. (PDF)
