# Supplementary material for: A cross-sectional study design to determine the prevalence of knowledge, attitude, and the preventive practice of food poisoning and its factors among postgraduate students in a public university in Selangor, Malaysia
Source: PLoS One. 2022 Jan 28;17(1):e0262313. doi: 10.1371/journal.pone.0262313 (PMC8797180; doi:10.1371/journal.pone.0262313)
Supplement: S1 Appendix — (PDF) [file pone.0262313.s001.pdf]

S1 Appendix: STROBE Checklist.

	Item No	Recommendation	Section
**Title and abstract**	1	(*a*) Indicate the study's design with a commonly used term in the title or the abstract	Title
		(*b*) Provide in the abstract an informative and balanced summary of what was done and what was found	Abstract
**Introduction**			
Background/ rationale	2	Explain the scientific background and rationale for the investigation being reported	Introduction, Para 1 & 2, 3, 4, 5, 6,
Objectives	3	State specific objectives, including any prespecified hypotheses	Introduction, Para 7
**Methods**			
Study design	4	Present key elements of study design early in the paper	Methods, Para 1 (Study design)
Setting	5	Describe the setting, locations, and relevant dates, including periods of recruitment, exposure, follow-up, and data collection	Methods, Para 1 (Study design)
Participants	6	(*a*) Give the eligibility criteria, and the sources and methods of selection of participants	Methods, Heading Participants
Variables	7	Clearly define all outcomes, exposures, predictors, potential confounders, and effect modifiers. Give diagnostic criteria, if applicable	Methods, Headings under operational definitions of outcomes 1,2,3 (outcomes) Headings under operational definition of exposures 1,2,3,4,5,6,7,8,9 (exposures, predictors, potential confounders, effect modifiers)
Data sources/ measurement	8*	For each variable of interest, give sources of data and details of methods of assessment (measurement). Describe comparability of assessment methods if there is more than one group	Methods, Para 1 & 2,3,4,5 (data sources/ measurements)
Bias	9	Describe any efforts to address potential sources of bias	Methods, Para 1 (Bias)
Study size	10	Explain how the study size was arrived at	Methods, Para 1, 2, 3 (Study size)
Quantitative variables	11	Explain how quantitative variables were handled in the analyses. If applicable, describe which groupings were chosen and why	Methods, Para 1 (Quantitative variables)
Statistical methods	12	(*a*) Describe all statistical methods, including those used to control for confounding	Methods, Para 1 (statistical methods)
		(*b*) Describe any methods used to examine subgroups and interactions	NA
		(*c*) Explain how missing data were addressed	No missing data
		(*d*) If applicable, describe analytical methods taking account of sampling strategy	NA

		(*e*) Describe any sensitivity analyses	NA
**Results**			
Participants	13*	(a) Report numbers of individuals at each stage of study—eg numbers potentially eligible, examined for eligibility, confirmed eligible, included in the study, completing follow-up, and analysed	Results,<br>Heading 1 (Participants)
		(b) Give reasons for non-participation at each stage	NA
		(c) Consider use of a flow diagram	NA
Descriptive data	14*	(a) Give characteristics of study participants (eg demographic, clinical, social) and information on exposures and potential confounders	Results,<br>Table 1, 2,3,4,5
		(b) Indicate number of participants with missing data for each variable of interest	NA
Outcome data	15*	Report numbers of outcome events or summary measures	Results,<br>Table 6,7,8,9,10,11,12,13,14,15,16
Main results	16	(*a*) Give unadjusted estimates and, if applicable, confounder-adjusted estimates and their precision (eg, 95% confidence interval). Make clear which confounders were adjusted for and why they were included	NA
		(*b*) Report category boundaries when continuous variables were categorized	Methods, Variables definitions
		(*c*) If relevant, consider translating estimates of relative risk into absolute risk for a meaningful time period	NA
Other analyses	17	Report other analyses done—eg analyses of subgroups and interactions, and sensitivity analyses	Statistical methods,<br>Para 1
**Discussion**			
Key results	18	Summarize key results with reference to study objectives	Discussion<br>Para 1-29
Limitations	19	Discuss limitations of the study, taking into account sources of potential bias or imprecision. Discuss both direction and magnitude of any potential bias	Discussion<br>Para 30
Interpretation	20	Give a cautious overall interpretation of results considering objectives, limitations, multiplicity of analyses, results from similar studies, and other relevant evidence	Discussion, para 31-33
Generalizability	21	Discuss the generalizability (external validity) of the study results	Generalizability of this study, para 1
**Other information**			
Funding	22	Give the source of funding and the role of the funders for the present study and, if applicable, for the original study on which the present article is based	Financial Disclosure,<br>Para 1
Order of Authors	23	Arhyel Buba Mshelia[1*], Malina Osman[2], Norashiqin Binti Misni[2]	Author<br>Byline
